# Comparing Nurse‐Led and Physician‐Led Bilevel Positive Airway Pressure Management in Hypercapnic Respiratory Failure: A Retrospective Cohort Study

**DOI:** 10.1002/nop2.70232

**Published:** 2025-04-22

**Authors:** Zhimin Zhao, Zhen Zhang, Xinlei Li, Bo Peng, Jinqian Xiong

**Affiliations:** ^1^ School of Medicine Henan Kaifeng College of Science Technology and Communication Kaifeng China; ^2^ Department of Respiratory and Critical Medicine The First Affiliated Hospital of Henan University Kaifeng China

**Keywords:** acute hypercapnic respiratory failure, BiPAP, clinical outcomes, non‐invasive ventilation, nurse‐led care, physician‐led care

## Abstract

**Aim:**

Bilevel positive airway pressure (BiPAP) management is a cornerstone in treating acute hypercapnic respiratory failure, with varying outcomes depending on the care model. This retrospective cohort study aims to compare the effectiveness of nurse‐led versus physician‐led BiPAP management in this context.

**Design:**

Retrospective cohort study.

**Methods:**

We analysed medical records of 189 patients with acute hypercapnic respiratory failure treated with BiPAP between January 2020 and December 2022. Patients were divided into nurse‐led (*n* = 101) and physician‐led (*n* = 88) groups based on their BiPAP management leadership. Outcomes measured included arterial blood gas (ABG) parameter improvements, intubation rates, length of hospital stay and 30‐day readmission rates. Statistical analysis involved chi‐squared tests, *t*‐tests, Mann–Whitney *U* tests and multivariable regression to adjust for confounders.

**Results:**

Both groups demonstrated significant improvements in ABG parameters, with no significant differences between groups. The nurse‐led group exhibited a lower intubation rate (9.9% vs. 21.6%, *p* = 0.026) and higher patient satisfaction scores (median 8 vs. 6, *p* < 0.001). Mortality rates, complication rates and 30‐day readmission rates were similar between the groups. Multivariable logistic regression confirmed the nurse‐led group had a lower likelihood of intubation (AOR: 0.401, *p* = 0.032) and higher patient satisfaction (AOR: 7.770, *p* < 0.001).

**Patient or Public Contribution:**

Our findings indicate that nurse‐led and physician‐led BiPAP management strategies for acute hypercapnic respiratory failure are equally effective in terms of ABG improvement, intubation rates, hospital stay length, and readmission rates. This suggests that with adequate training and supervision, nurse‐led BiPAP management can be as effective as physician‐led management, offering a viable alternative in healthcare settings where physician resources are limited.

## 1 Introduction

Bilevel Positive Airway Pressure (BiPAP) is increasingly recognised as a vital non‐invasive ventilation (NIV) strategy in the management of acute hypercapnic respiratory failure, a condition marked by an elevated level of carbon dioxide in the blood (Tobias 2000; Comellini et al. 2019). NIV, including BiPAP, has been extensively documented to alleviate symptoms of acute respiratory failure, enhance gas exchange and reduce the need for invasive mechanical ventilation (Tobias 2000; Mas and Masip 2014; Osadnik et al. 2017). BiPAP management has emerged as a crucial intervention for acute hypercapnic respiratory failure, particularly given the variability in outcomes associated with different care models (Osadnik et al. 2017). While BiPAP's effectiveness is well established, the comparative efficiency of differing management strategies—particularly nurse‐led versus physician‐led care—remains underexplored, though it is suggested that nurse‐led interventions may be as effective as physician‐led ones in certain diseases and could potentially lead to improvements in patient management and satisfaction (Kuethe et al. 2013; Wang et al. 2018).

Acute hypercapnic respiratory failure, characterised by elevated arterial carbon dioxide levels, often necessitates comprehensive management strategies to optimise patient outcomes (Osadnik et al. 2017). It is primarily seen in conditions such as chronic obstructive pulmonary disease (COPD), and managing this acute condition effectively is crucial to reduce morbidity and mortality (Meservey et al. 2020). The literature suggests that a well‐structured BiPAP protocol can reduce the need for invasive mechanical ventilation and its associated complications, including intubation‐related injuries and hospital‐acquired infections (Osadnik et al. 2017). Previous studies have illustrated that the effectiveness of BiPAP may depend significantly on the clinical setting and the healthcare professionals' expertise (Cabrini et al. 2015). Given the growing emphasis on cost‐effectiveness and resource allocation in healthcare, exploring alternative management strategies, such as nurse‐led care, could provide valuable insights into optimising patient care delivery (Ndosi et al. 2014).

In recent years, healthcare systems have faced significant strain, necessitating more flexible staffing models while maintaining high‐quality patient care (Wang et al. 2018; Ndosi et al. 2014; Lai et al. 2018). This has led to the exploration of nurse‐led models in various clinical settings. However, the application of nurse‐led models in acute care settings, particularly in the management of acute hypercapnic respiratory failure with BiPAP, is not well documented.

This retrospective cohort study analyses data from patients with acute hypercapnic respiratory failure who received BiPAP under either nurse‐led or physician‐led protocols. By examining outcomes from these two groups, this research aims to assess the practical equivalency of nurse‐led care in situations where physician resources are scarce. Our analysis seeks to provide evidence that might support nurse‐led BiPAP management as a competent alternative to physician‐led care, thereby informing future staffing models and potentially guiding policy decisions in healthcare systems.

## 2 Methods

### 2.1 Study Design

This retrospective cohort study evaluated the effectiveness of nurse‐led versus physician‐led BiPAP management in patients with acute hypercapnic respiratory failure. We reviewed the medical records of 189 patients treated with BiPAP from January 2020 to December 2022 at the Intensive Care Unit (ICU) and Department of Respiratory and Critical Care Medicine in our hospital. The study was approved by the Ethics Committee of REDACTED (approval number 2023‐03‐044). Due to the retrospective nature of the study, the requirement for informed consent was waived. All patient data were anonymized and de‐identified prior to analysis to ensure confidentiality and compliance with ethical standards.

### 2.2 Study Population

Patients included in this study were those aged 18 years and above with a confirmed diagnosis of acute hypercapnic respiratory failure, indicated by arterial blood gases (ABGs) showing a partial pressure of carbon dioxide (PaCO_2_) greater than 45 mmHg. All patients were required to have received BiPAP therapy during the designated study period as part of their treatment regimen. The primary causes of acute hypercapnic respiratory failure included acute exacerbation of chronic obstructive pulmonary disease (AECOPD), obstructive sleep apnea‐hypopnea syndrome (OSAHS), severe pneumonia with acute respiratory failure and other diagnoses such as pulmonary embolism and interstitial lung disease exacerbation.

Exclusion criteria were carefully considered to maintain the integrity of the study and ensure that the results specifically addressed the research question. Patients were excluded if they had a primary diagnosis other than acute respiratory failure, such as cardiac failure or neuromuscular diseases, which could independently affect the outcomes of BiPAP therapy. Additionally, patients who had undergone invasive mechanical ventilation or had a tracheostomy before the initiation of BiPAP therapy were excluded to prevent confounding treatment effects. The study also excluded patients with incomplete medical records or missing initial or follow‐up ABG data, which are crucial for assessing the treatment's efficacy. Furthermore, patients with do not intubate or do not resuscitate orders at the time of admission were excluded to avoid ethical and treatment complexities that could skew the study outcomes.

### 2.3 BiPAP Management Protocols

The nurse‐led protocol involved registered respiratory nurses with specialised training in NIV management. These nurses managed the initiation and adjustment of BiPAP settings according to the clinical protocol designed to optimise patient ventilation. The protocol specified the use of S/T mode, with initial inspiratory positive airway pressure (IPAP) and expiratory positive airway pressure (EPAP) settings determined based on the patient's underlying disease, ABG parameters, and individual tolerance. Specifically, IPAP was set between 8 and 12 cmH_2_O, and EPAP was set between 4 and 6 cmH_2_O. These settings were gradually increased over 2–20 min to achieve an appropriate therapeutic level, with a target pressure support of at least 10 cmH_2_O. After 30 min of ventilation, the effectiveness and patient comfort were assessed. If tolerated by the patient and oxygen saturation (SpO_2_) levels were maintained above 90%, IPAP was incrementally titrated in 2 cmH_2_O increments, within a range of 12–20 cmH_2_O, based on patient tolerance and the degree of PaCO_2_ reduction. The respiratory rate was set at 16 breaths per minute, oxygen flow rates adjusted between 3 and 8 L/min. Nurses closely monitored patients throughout the treatment to ensure safety and efficacy of the ventilation. This protocol was collaboratively developed by the hospital's Respiratory Therapy Department in conjunction with the ICU multidisciplinary team, comprising respiratory physicians, senior nursing staff and clinical respiratory specialists, ensuring it was evidence‐based and tailored to the hospital's specific patient population and resource availability. Furthermore, the protocol incorporates ventilator waveform interpretation and the detection of patient‐ventilator asynchrony, with nurses receiving specialised training to read and interpret pressure–time and flow‐time curves. This training enabled them to identify signs of asynchrony, such as ineffective trigger attempts, double triggering or insufficient flow, which are visible in the pressure, flow and volume waveforms displayed on the BiPAP interface screen. In addition to waveform analysis, nurses closely monitor clinical signs that may indicate unresolved asynchrony, such as the use of accessory muscles, agitation or tachypnea, which often suggest that the patient's clinical status is not adequately addressed by the current ventilation settings, indicating a mismatch with the ventilator. When asynchrony is detected, the nurse‐led protocol typically recommends specific interventions, including adjusting inspiratory trigger sensitivity, modifying rise time, or altering IPAP/EPAP settings to optimise patient–ventilator interaction. In cases of persistent or severe asynchrony, the protocol advises nurses to immediately consult with respiratory therapists or physicians to assess whether further adjustments or interventions are necessary.

Conversely, the physician‐led protocol was directed entirely by respiratory physicians who took full responsibility for all decisions regarding the BiPAP settings and adjustments. These physicians performed daily evaluations of each patient, modifying the BiPAP treatment plans based on a comprehensive analysis that combined the results of ABG measurements with other clinical assessments. Clinical assessment parameters included monitoring vital signs such as pulse, blood pressure and respiratory rate, as well as evaluating respiratory patterns and chest wall movement. In addition to these assessments, physicians also reviewed ventilator waveforms, including pressure–time and flow–time curves, to detect patient–ventilator asynchrony and assess the adequacy of ventilatory support. Neurological status was monitored through assessments of consciousness level, such as using the Glasgow Coma Scale or simple evaluations of alertness and response to stimuli, to detect any potential respiratory or central nervous system deterioration.

### 2.4 Data Collection

Data were collected on patient demographics, clinical characteristics, BiPAP settings and treatment outcomes. Primary outcomes measured were improvements in ABG parameters, particularly the reduction in partial pressure of carbon dioxide (PaCO_2_), assessed at baseline and 24 h post‐treatment. Secondary outcomes included pH, intubation rates, length of hospital stay, 30‐day readmission rates, NIV time, symptom relief, occurrence of complications and mortality. These secondary outcomes were monitored continuously throughout the hospital stay and during the 30‐day follow‐up period. At discharge, patient satisfaction was evaluated using a hospital‐designed nursing satisfaction scale. This scale ranges from 0 to 10, where a score of 0–3 indicates dissatisfaction, 4–7 indicates moderate satisfaction, and 8–10 signifies high satisfaction.

### 2.5 Statistical Analysis

Analyses between the two groups were performed using chi‐squared tests or Fisher's exact test for categorical data and independent *t*‐tests for continuous data that followed a normal distribution. The Mann–Whitney *U* test was applied to continuous variables that did not meet the assumption of normality, as assessed by the Shapiro–Wilk test. Effect sizes for significant findings were reported using Cohen's *d* for *t*‐tests and *r* for Mann–Whitney *U* tests to provide insight into the magnitude of differences observed. Multivariable logistic regression was utilised to adjust for potential confounders including age, sex, comorbidities and baseline severity of the disease, ensuring a robust model for assessing the impact of management type on outcomes. The variables included in the logistic model were chosen based on their theoretical relevance and statistical significance in univariate analyses. A *p*‐value of less than 0.05 was considered statisticallysignificant.

## 3 Results

### 3.1 Demographic and Clinical Characteristics

A total of 189 patients were included in this study, with 101 in the nurse‐led group and 88 in the physician‐led group. Both groups were predominantly male (80.2% vs. 76.1%; *p* = 0.499) with similar ages (67.55 ± 7.56 years vs. 67.84 ± 8.06 years; *p* = 0.801). Smoking status was slightly higher in the nurse‐led group (73.3% vs. 62.5%; *p* = 0.113), but there were no significant differences in aetiology distribution and comorbid conditions including bronchiectasis, pulmonary hypertension, severe pneumonia, chronic obstructive pulmonary disease, diabetes, coronary heart disease and heart failure between the groups. Clinical measurements such as systolic and diastolic blood pressure, heart rate and respiratory rate also showed no significant differences (Table 1). In the nurse‐led group, 7 cases (6.9%) required physician intervention due to unresolved patient‐ventilator asynchrony.

**TABLE 1:** Demographic and clinical characteristics of the two groups.

Characteristics	Nurse‐led group (*n* = 101)	Physician‐led group (*n* = 88)	*P*
Male, *n* (%)	81 (80.2%)	67 (76.1%)	0.499
Age (years)	67.55 ± 7.56	67.84 ± 8.06	0.801
BMI (kg/m^2^)	26.91 ± 4.41	26.19 ± 4.53	0.271
Smoking, *n* (%)	74 (73.3)	55 (62.5)	0.113
Aetiology, *n* (%)			
AECOPD	34 (33.7)	37 (42.0)	0.589
OSAHS	7 (6.9)	5 (5.7)	
Severe pneumonia with acute respiratory failure	17 (16.8)	19 (21.6)	
Pulmonary embolism	7 (6.9)	3 (3.4)	
Interstitial lung disease exacerbation	2 (2.0)	0	
Pulmonary infection	29 (28.7)	20 (22.7)	
Heart failure	5 (5.0)	4 (4.5)	
Comorbidities, *n* (%)			
Bronchiectasis	19 (18.8)	20 (22.7)	0.507
Pulmonary hypertension	17 (16.8)	13 (14.8)	0.699
Severe pneumonia	19 (18.8)	22 (25.0)	0.303
COPD	36 (35.6)	38 (43.2)	0.290
Diabetes	37 (36.6)	26 (29.5)	0.302
Coronary heart disease	36 (35.6)	34 (38.6)	0.671
Heart failure	17 (16.8)	15 (17.0)	0.969
SBP (mmHg)	133.79 ± 14.23	130.69 ± 12.91	0.363
DBP (mmHg)	87.23 ± 9.07	85.24 ± 10.04	0.154
Heart rate (bpm)	114.62 ± 12.85	113.90 ± 10.89	0.678
Respiratory rate (bpm)	33 (31, 34)	32 (31, 34)	0.718
SAPS II score	31.51 ± 7.44	32.47 ± 7.47	0.383
APACHE II score	15.79 ± 3.80	16.18 ± 4.14	0.501

Abbreviations: AECOPD, acute exacerbation of chronic obstructive pulmonary disease; APACHE II, Acute Physiology and Chronic Health Evaluation; bpm, beats per minute; COPD, chronic obstructive pulmonary disease; DBP, diastolic blood pressure; OSAHS, obstructive sleep apnea‐hypopnea syndrome; SAPS II, simplified acute physiology score; SBP, systolic blood pressure.

### 3.2 Clinical Outcomes

After intervention, the length of hospital stay and NIV time were similar between the two groups. Symptom relief was achieved in a median of 6 days in both groups (*p* = 0.407). A significant difference was observed in the requirement for endotracheal intubation, with the nurse‐led group showing a notably lower rate (9.9% vs. 21.6%; *p* = 0.026). Mortality rates did not differ significantly between the two groups (5% vs. 10.2%; *p* = 0.167). Complications such as pneumonia, sepsis, gastrointestinal disorders, myocardial infarction and multiple pneumothoraxes were similarly distributed between the groups, with no significant differences. The 30‐day readmission rate was also not significantly different, with 5.9% in the nurse‐led group and 12.5% in the physician‐led group (*p* = 0.116). Notably, patient satisfaction scores were significantly higher in the nurse‐led group, with a median score of 8 compared to 6 in the physician‐led group (*p* < 0.001) (Table 2).

**TABLE 2:** Outcomes comparison between two groups.

Outcomes	Nurse‐led group (*n* = 101)	Physician‐led group (*n* = 88)	*p*
Length of hospital stay (days)	11.0 (8.5, 14.0)	10. 0 (8.0, 13.0)	0.271
Non‐invasive ventilation time (days)	9. 0 (5.5, 11.0)	8. 0 (5.0, 11.0)	0.538
Symptom relief time (days)	6. 0 (3.5, 9.0)	6. 0 (3.0, 8.0)	0.407
Need for endotracheal intubation, *n* (%)	10 (9.9)	19 (21.6)	0.026
Mortality, *n* (%)	5 (5.0)	9 (10.2)	0.167
Complications, *n* (%)	31 (30.7)	31 (35.2)	0.508
Pneumonia	16 (15.8)	19 (21.6)	0.310
Sepsis	3 (3.0)	7 (8.0)	0.230
Gastrointestinal tract disorders	6 (5.9)	5 (5.7)	0.940
Myocardial infarction	5 (5.0)	5 (5.7)	0.823
Multiple pneumothoraxes	3 (3.0)	0 (0.0)	0.250
30‐day readmission, *n* (%)	6 (5.9)	11 (12.5)	0.116
Patient satisfaction	8.0 (7.0, 9.0)	6. 0 (5.0, 7.0)	0.000

### 3.3 Blood Gas Analysis and pH

Both groups showed significant improvement in ABGs and pH from pre‐treatment to 24 h post‐treatment. In the nurse‐led group, the mean PaO_2_ improved from 53.83 ± 9.13 mmHg to 80.15 ± 13.15 mmHg (*p* < 0.001), and in the physician‐led group from 55.80 ± 9.61 mmHg to 81.60 ± 9.10 mmHg (*p* < 0.001). The PaCO_2_ also decreased significantly in both groups (both *p* < 0.001). Similarly, improvements in the PaO_2_/FiO_2_ ratio and pH were noted in both groups (all *p* < 0.001), indicating effective BiPAP management in both nurse‐led and physician‐led settings. However, there were no significant differences in ABGs and pH between the groups either before or after the treatment (all *p* > 0.05) (Table 3).

**TABLE 3:** Comparison of blood gas analysis and pH between two groups before and after treatment.

Outcomes	Nurse‐led group (*n* = 101)			Physician‐led group (*n* = 88)		
	Pre‐treatment	24 h post‐treatment	*p*	Pre‐treatment	24 h post‐treatment	*p*
PaO_2_	53.83 ± 9.13	80.15 ± 13.15	< 0.001	55.80 ± 9.61	81.60 ± 9.10	< 0.001
PaCO_2_	69.94 ± 5.50	54.25 ± 6.19	< 0.001	69.18 ± 4.69	53.27 ± 6.19	< 0.001
PaO_2_/FiO_2_	206.26 ± 26.21	299.30 ± 36.12	< 0.001	210.08 ± 27.74	300.52 ± 37.98	< 0.001
pH	7.26 ± 0.05	7.39 ± 0.03	< 0.001	7.26 ± 0.04	7.40 ± 0.03	< 0.001

### 3.4 Logistic Regression Analysis

The logistic regression analysis assessed various factors of patient intubation and nursing satisfaction in patients with acute hypercapnic respiratory failure managed with BiPAP. In the multivariate analysis, the nurse‐led group showed a significantly lower likelihood of requiring endotracheal intubation compared to the physician‐led group (adjusted odds ratio [AOR]: 0.401; 95% CI: 0.174–0.926; *p* = 0.032). Additionally, the presence of coronary heart disease emerged as a significant predictor of poorer outcomes, increasing the risk of requiring endotracheal intubation (AOR: 2.404; 95% CI: 1.066–5.421; *p* = 0.034). Patient satisfaction also differed markedly between groups, with those in the nurse‐led group significantly more likely to report higher satisfaction (AOR: 7.770; 95% CI: 3.966–15.219; *p* < 0.001). Furthermore, the baseline SAPS II score was also linked to patient satisfaction, with higher baseline scores predicting lower satisfaction rates (AOR: 1.051; 95% CI: 1.005–1.098; *p* = 0.029) (Table 4).

**TABLE 4:** Logistic regression analysis of outcomes.

Outcomes	Characteristics	Univariate analysis		Multivariate analysis	
		Odds ratio (95% CI)	*p*	Adjusted odds ratio (95% CI)	*p*
Need for endotracheal intubation	Nurse‐led	0. 235 (0.085, 0.652)	0.005	0. 401 (0.174, 0.926)	0.032
	Physician‐led	Reference		Reference	
	Coronary heart disease	2. 686 (1.081, 6.671)	0.033	2.404 (1.066, 5.421)	0.034
Patient Satisfaction ≥ 8	Nurse‐led	8. 562 (4.078, 17.976)	< 0.001	7.770 (3.966, 15.219)	< 0.001
	Physician‐led	Reference		Reference	
	SAPS II score	1. 055 (1.005, 1.108)	0.031	1. 051 (1.005, 1.098)	0.029

Abbreviation: SAPS II, Simplified Acute Physiology Score.

## 4 Discussion

This retrospective cohort study compared nurse‐led versus physician‐led BiPAP management in patients with acute hypercapnic respiratory failure. Overall, our findings suggest that both approaches are equally effective in managing this condition, as evidenced by similar outcomes in ABGs, intubation rates, and hospital length of stay between the two groups. These results support the premise that non‐physician clinicians, when appropriately trained and guided by standardised protocols, can effectively manage complex respiratory therapies, which is an important consideration in healthcare settings with limited physician availability.

Notably, the nurse‐led group in our study demonstrated a lower rate of endotracheal intubation compared to the physician‐led group. While this may initially appear counterintuitive, it aligns with previous research suggesting that protocol‐driven, nurse‐led interventions can yield outcomes similar to or better than physician‐led care (Kuethe et al. 2013; Htay and Whitehead 2021; Hirzallah et al. 2019). One plausible explanation is that nurses often maintain a continuous bedside presence, enabling early detection of clinical deteriorations and timely adjustments to BiPAP parameters. The structured protocols and regular patient monitoring are pivotal in managing dynamic conditions like acute hypercapnic respiratory failure (Gupta et al. 2021). This aligns with findings from a previous study, which highlighted the effectiveness of a nurse‐driven protocol in reducing the intubation rate to 15% among patients with acute hypercapnic respiratory failure (Contou et al. 2013). Moreover, the collaborative and protocol‐driven nature of nurse‐led models facilitates more frequent multidisciplinary discussions, which in turn promote timely non‐invasive interventions before resorting to intubation. For example, evidence suggests that nurse‐driven protocols incorporating regular interdisciplinary team input enhance clinical decision‐making and patient outcomes, particularly in the management of acute respiratory failure (Cassibba et al. 2024). However, it is important to note that not all studies have found nurse‐led management to yield superior results compared to physician‐led outcomes; some studies have highlighted the context‐dependent efficacy of nurse‐led models, emphasising the need for appropriate patient selection and robust implementation frameworks to optimise their effectiveness (Kuethe et al. 2013; Duyndam et al. 2020).

On the other hand, the presence of coronary heart disease was identified as a predictor of poorer outcomes, including increased intubation risk, underscoring the need for specialised interventions in this subgroup. Interestingly, higher baseline SAPS II scores were correlated with greater satisfaction, suggesting that patients with more severe initial conditions might perceive greater value in attentive and comprehensive care. In the present study, patient satisfaction was significantly higher in the nurse‐led group. This finding could reflect the continuous patient engagement and perhaps a more consistent presence that nurses have at the bedside compared to physicians, which has been shown to contribute positively to patient perceptions of care quality and satisfaction (Chan et al. 2018; Garner et al. 2017). A study found that nurse‐led pain management programmes led to substantial improvements in patient satisfaction, especially in terms of staff responsiveness and communication (Germossa et al. 2022). Similarly, another study observed that nurse‐led communication interventions, such as interprofessional bedside rounds, improved patient satisfaction by enhancing the quality of communication and collaboration between healthcare providers (Austin et al. 2021). These aspects of care, particularly when nurses provide consistent and compassionate communication, have been shown to improve patient outcomes and overall satisfaction with their care experience.

Our analysis did not reveal significant differences in the primary outcome of ABG improvement post‐BiPAP between the two groups. Both groups showed substantial improvements in PaO_2_ and PaCO_2_ levels, underscoring the efficacy of BiPAP as a critical intervention for acute hypercapnic respiratory failure. These results are consistent with existing literature that documents the benefits of BiPAP in enhancing gas exchange and reducing the need for more invasive interventions (Osadnik et al. 2017; Akhter and Rizvi 2017). However, most cited studies and our own findings are limited by relatively short follow‐up intervals. Therefore, the long‐term effects of nurse‐led versus physician‐led management strategies on patient recovery, functional status, and quality of life remain largely unexplored.

While our study highlights promising aspects of nurse‐led BiPAP management, it must be contextualised within the broader literature. Particularly in high‐acuity environments, differences in training, institutional support, and case complexity can significantly influence outcomes, sometimes resulting in less favourable or inconsistent findings for nurse‐led interventions (Kuethe et al. 2013; Duyndam et al. 2020; Balfour 2020). Variability in patient acuity, case complexity and institutional protocols is frequently cited as a factor contributing to the heterogeneity in reported outcomes (D'Cruz et al. 2023; Stevens et al. 2024). Effective nurse‐led care hinges on robust training programmes, adherence to standardised protocols, and structured competency assessments. However, in resource‐limited settings, establishing such infrastructure can be particularly challenging. Variability in the availability and quality of training contributes to inconsistent results, underscoring the need for ongoing professional development, simulation‐based learning and periodic competency reassessments (Balfour 2020; Guilhermino et al. 2018). Economic evaluations are also essential to determine the cost‐effectiveness of nurse‐led BiPAP management, particularly in resource‐limited systems. Studies have indicated that nurse‐led models can optimise resource utilisation, reduce healthcare costs and improve care quality and outcomes (Ora et al. 2023; Al Achkar and Chaaban 2025). Additionally, institutional policies, regulatory frameworks and interdisciplinary collaboration are crucial for clearly defining the roles and responsibilities of nurses in respiratory management. Studies highlight that well‐structured institutional support fosters effective nurse‐led interventions by integrating interdisciplinary efforts and aligning responsibilities with regulatory standards (Dosher et al. 2014; Dammeyer et al. 2012; Yorke et al. 2017). Without sufficient institutional backing and well‐defined role delineation, the adoption of nurse‐led models may be hindered, especially in complex cases that require advanced diagnostic and therapeutic decision‐making capabilities (Yorke et al. 2017; Conway and Richardson 2004). Moreover, high nurse‐to‐patient ratios and escalating workloads can compromise the effectiveness of care delivery and lead to burnout, while staff turnover exacerbates these challenges by disrupting continuity of care (Maxie 2025; Vozzella and Hehman 2023). Such challenges emphasise the need for a cohesive framework that prioritises collaborative practice and strategic role distribution in respiratory care. Another crucial consideration is the long‐term impact of nurse‐led care in BiPAP management. While our study highlights the short‐term efficacy of nurse‐led BiPAP interventions, further research is necessary to explore long‐term outcomes. Evidence suggests that structured nurse‐driven protocols can significantly impact ventilatory management and patient outcomes over extended periods by ensuring consistent, protocol‐based interventions (Cassibba et al. 2025).

Despite these challenges, our findings underscore the potential value of nurse‐led BiPAP management, particularly in settings where physician resources are limited. By reallocating physician time to the most complex cases and empowering trained nurses to manage stable patients under established protocols, healthcare systems can improve efficiency and maintain care quality. Studies suggest that such nurse‐led models can optimise resource utilisation, reduce physician workload and burnout, while simultaneously increasing nurse autonomy and job satisfaction (Matlhola 2020). However, the lack of standardised training and clear role delineation in some settings may hinder the full integration of nurse‐led interventions, especially in complex cases requiring advanced diagnostic and therapeutic decision‐making (Matlhola 2020; Guilhermino 2018). In rural and underserved areas, expanding nurses' roles in respiratory care can improve access to treatment, address healthcare disparities, and enhance the timeliness of interventions. Furthermore, nurse‐led models have demonstrated cost‐effectiveness, offering significant operational savings while maintaining or even improving clinical outcomes, which is especially critical for resource‐constrained systems (Randall et al. 2017).

Our study has limitations. The retrospective design and single‐centre setting is inherently susceptible to bias, including selection bias and reliance on the accuracy of medical records. Variations in institutional protocols, training programmes and adherence to BiPAP guidelines may influence the observed outcomes, limiting generalisability to other healthcare settings. Future research should employ prospective designs or randomised designs to mitigate these biases and assess how institutional and resource variations affect the success of nurse‐led models. Efforts to expand training programmes, strengthen institutional support and investigate cost‐effectiveness and resource utilisation will be key to broader implementation. Moreover, future work should focus on long‐term outcomes, exploring whether nurse‐led strategies influence patient survival, readmission rates and quality of life beyond the acute phase.

## 5 Conclusion

In conclusion, our findings suggest that nurse‐led BiPAP management strategies hold promise for broader implementation in suitable patient populations, offering a potential solution to alleviate physician resource constraints. By further investigating the comparative effectiveness and long‐term implications of nurse‐led care, healthcare systems can optimise resource allocation and improve patient outcomes in the management of acute hypercapnic respiratory failure.

## Author Contributions

Z.Z. performed the study concepts, study design, manuscript preparation and manuscript editing. Z.Z. and X.L. contributed to the quality control of data and algorithms, data analysis and interpretation. B.P. and J.X. contributed to data acquisition and statistical analysis. Z.Z. also supervised and reviewed the manuscript. All authors read and approved the final manuscript.

## Ethics Statement

The study was approved by the Ethics Committee of the First Affiliated Hospital of Henan University (approval number 2023‐03‐044). Due to the retrospective nature of the study, the requirement for informed consent was waived. All patient data were anonymised and de‐identified prior to analysis to ensure confidentiality and compliance with ethical standards.

## Consent

The authors have nothing to report.

## Conflicts of Interest

The authors declare no conflicts of interest.

## Data Availability

The datasets generated and/or analysed during the current study are available from the corresponding author upon reasonable request, subject to privacy and ethical restrictions.
